# Impact of Complications and Comorbidities on the Intensive Care Length of Stay after Aneurysmal Subarachnoid Haemorrhage

**DOI:** 10.1038/s41598-020-63298-9

**Published:** 2020-04-10

**Authors:** Alexander Hammer, Gholamreza Ranaie, Frank Erbguth, Matthias Hohenhaus, Martin Wenzl, Monika Killer-Oberpfalzer, Hans-Herbert Steiner, Hendrik Janssen

**Affiliations:** 1 Department of Neurosurgery, Paracelsus Medical University, Breslauer Str. 201, 90471 Nuremberg, Bavaria Germany; 2 Department of Neurology, Paracelsus Medical University, Breslauer Str. 201, 90471 Nuremberg, Bavaria Germany; 3 Department of Anaesthesiology, Paracelsus Medical University, Breslauer Str. 201, 90471 Nuremberg, Bavaria Germany; 4 Paracelsus Medical University, Neurology/Research Institute of Neurointervention, Ignaz Harrer Str. 79, Salzburg, Austria; 5 Department of Neuroradiology, Ingolstadt General Hospital, Krumenauerstraße 25, 85049 Ingolstadt, Bavaria Germany

**Keywords:** Stroke, Risk factors

## Abstract

In this observational study, we analysed a cohort of 164 subarachnoid haemorrhage survivors (until discharge from intensive care) with the aim to detect factors that influence the length of stay (LOS) in intensive care with multiple linear regression methods. Moreover, binary logistic regression methods were used to examine whether the time in intensive care is a predictor of outcome after 1 year. The clinical 1-year outcome was measured prospectively in a 12-month follow-up by telephone interview and categorised by the modified Rankin Scale (mRS). Patients who died during their stay in intensive care were excluded. Complications like pneumonia (β = 5.11; 95% CI = 1.75–8.46; p = 0.0031), sepsis (β = 9.54; 95% CI = 3.27–15.82; p = 0.0031), hydrocephalus (β = 4.63; 95% CI = 1.82–7.45; p = 0.0014), and delayed cerebral ischemia (DCI) (β = 3.38; 95% CI = 0.19–6.56; p = 0.038) were critical factors depending the LOS in intensive care as well as decompressive craniectomy (β = 5.02; 95% CI = 1.35–8.70; p = 0.0077). All analysed comorbidities such as hypertension, diabetes, hypothyroidism, cholesterinemia, and smoking history had no significant impact on the LOS in intensive care. LOS in intensive care (OR = 1.09; 95% CI = 1.03–1.15; p = 0.0023) as well as WFNS grade (OR = 3.72; 95% CI = 2.23–6.21; p < 0.0001) and age (OR = 1.06; 95% CI = 1.02–1.10; p = 0.0061) were significant factors that had an impact on the outcome after 1 year. Complications in intensive care but not comorbidities are associated with higher LOS in intensive care. LOS in intensive care is a modest but significant predictor of outcomes after subarachnoid haemorrhage.

## Introduction

Subarachnoid haemorrhage (SAH) is a devastating disease that challenges all treating disciplines. Aside from direct effects of SAH on the brain parenchyma, and directly associated complications like rebleeding, delayed cerebral ischemia, and hydrocephalus, there is evidence that further medical complications play a crucial role in the outcome after SAH^1,2^. It was reported that the proportion of deaths from medical complications is comparable to the proportion of deaths attributed to the direct effects of the initial haemorrhage, rebleeding, and vasospasm^1,2^. However, these complications might be preventable or at least controllable. A better outcome might be achievable with improved intensive care management^1^. Beside high rates of morbidity and mortality, SAH is associated with prolonged ICU length of stay (LOS)^2–4^. Initial poor clinical condition and treatment modality, but especially duration of stay in ICU, are substantial economic factors in SAH. Treatment costs of SAH in the first year easily exceed costs in the first year of ischemic stroke^5,6^.

Some nosocomial infections in SAH are known to determine prolonged LOS^3,7^. Galea *et al*. reported that patients who had significantly longer LOS after SAH had unfavourable outcomes at discharge^8^. However, it is uncertain whether the total LOS in ICU is a direct predictor of outcome.

This study aimed to investigate factors that determine the LOS in intensive care like complications, comorbidities, and interventions after SAH. Furthermore, we evaluated the direct impact of the LOS in intensive care on the outcome after suffering from SAH.

## Methods

Data were extracted and analysed from an anonymised observational database called “SAB-Datenbank Nürnberg” comprising 203 cases of ruptured intracranial aneurysms from 2012 to 2017 which were treated by the departments of neuroradiology, neurology, anaesthesiology and neurosurgery at the Paracelsus Medical University Nuremberg. The database is stored at a local hard-disk at the Department of Neurosurgery of the Paracelsus Medical University and is contemporary not publicly accessible for reasons of further analysis and publications. Data generated or analysed particularly for this study are all included in this published article.

Our local institutional review board stated, that for this study no submission to the ethics committee is necessary (Ethics-Comitee of the Bavarian Chamber of Physicians; 2019-124 Dr. AB/Gu). Informed consent of the patients or their relatives was obtained during the initial hospital stay or during the telephone interview in the follow up. The study was performed in accordance with the ethical standards laid down in the 1964 declaration of Helsinki and its later amendments. Data from the above-mentioned database were used in parts for previous publications^9–11^.

Between 2012 and 2017, we observed 164 cases of SAH for this study that met the following criteria for inclusion:

1. Time between aneurysm rupture and treatment <48 hours.
2. Informed consent from the patient, a patient’s relative, or the patient’s guardian.
3. Verification of SAH with cranial CT or lumbar puncture and verification of an associated intracranial aneurysm diagnosed in most cases by digital subtraction angiography or, alternatively, by CT angiography if an immediate operation had to be performed.
4. Patient survival during initial hospital stay.

The decision for the allocation of the treatment modality was part of the standard care of the patients and was done by specialised neurosurgeons and neuroradiologists in consensus. The patients were either treated by microsurgical clipping or endovascular procedures comprising sole coiling, coiling in combination with balloon or stent-assisted remodelling, or the use of endovascular or intrasaccular flow-diverters.

The clinical outcome was measured prospectively in a 12-month follow-up by telephone interview and categorised by the modified Rankin Scale (mRS). The mRS was applied for measuring the degree of disability or dependence in the daily activities of patients after suffering from stroke (including SAH) or other causes of neurological disability. The scale reaches from 0 to 6 with the following meanings: 0 - No symptoms; 1 - No significant disability; 2 - Slight disability; 3 - Moderate disability; 4 - Moderately severe disability; 5 - Severe disability; 6 - Dead^12,13^. Usage of the 1-year-mRS correspond with the recommendations of the multidisciplinary research group of Stienen *et al*.^14^.

For this study, we collected and analysed all available data concerning pre-defined comorbidities and history of smoking as well as complications and interventions.

The comorbidities hypertension, diabetes mellitus, hypothyroidism, hypercholesterolemia, and history of smoking as well as the complications and interventions pneumonia, sepsis, hydrocephalus, diabetes insipidus, decompressive craniectomy, early cerebral ischemia, and delayed cerebral ischemia were used for examination in this study.

We collected all available data concerning the presence of the comorbidities hypertension, diabetes mellitus, hypothyroidism and hypercholesterolemia on patients admission and documented the data in the patients file. Analysis of this data for the purpose of this study was done retrospectively. Smoking was only counted if patients were current smokers. If smoking was quit in the past patients were not considered as smokers. For this purpose all available medical records were analysed^9^.

Experienced anaesthetists diagnosed the complications pneumonia and sepsis according to the following criteria:

Pneumonia is defined according to the American College of Chest Physicians criteria in patients who had a new infiltrate on chest x-ray plus two of the three following conditions: elevated white blood cell count more than 10,000 cells/ml, fever, or purulent sputum.

Sepsis is defined as a life-threatening organ dysfunction due to a dysregulated host response to infection. Assessment of sepsis conformed to the SOFA (Sepsis-related Organ Failure Assessment) criteria in order to describe organ dysfunction/failure^15^.

Hydrocephalus was defined as an active distension of the ventricular system of the brain resulting from inadequate passage of cerebrospinal fluid from its point of production within the cerebral ventricles to its point of absorption into the systemic circulation caused by SAH^16^. For diagnosis of hydrocephalus neurosurgeons, neurologists and radiologists considered clinical criteria as well as diagnostic imaging. In cases of hydrocephalus neurosurgeons implanted an external ventricular drainage. Chronic hydrocephalus was treated eventually by a ventricular-peritoneal shunting system. Raised intracranial pressure caused by general brain edema, intracerebral haemorrhage, or cerebral ischemia, which was not sufficiently controllable by conservative intensive care treatment methods, resulted in decompressive craniectomy. Diagnostic imaging and clinical parameters were grounds for the indication to decompress. Decompressive hemicraniectomy, bifrontal craniectomy, and decompression of the posterior cranial fossa were the decompression techniques in our cohort.

A clinically apparent new ischemia within the first 3 days after treatment (detectable in diagnostic imaging) was defined as early cerebral ischemia (ECI). All events after 3 days were defined as DCI. DCI is defined by the inclusion of a focal (hemiparesis, aphasia, hemianopia, or neglect) or global neurological impairment lasting for at least 1 hour and/or cerebral infarction, which is not apparent immediately after aneurysm treatment and cannot be attributed to other causes^17^. In our collective DCI was always associated with cerebral infarction after 3 days.

### Statistics

For descriptive statistics (frequencies), multiple linear regression, and binary logistic regression analysis, we used SPSS version 21 (SPSS Inc., Cary, SC).

For analysis of the dependence of the LOS in ICU, we performed multiple linear regression with a maximum number of 10 independent variables. We included comorbidities, complications, and interventions that are in line with a possible pathophysiological hypothesis defined by the authors, which could explain a possible impact on the LOS in ICU. The least number of cases per independent variable was 7. We adjusted the multiple linear regression analysis with the baseline data sex, age, and WFNS grade. We used the Analysis of Variance (ANOVA) approach for testing the overall significance of the observed multiple regression (F -test). We report R^2^, adjusted R^2^, and the Durbin-Watson coefficient.

P-values below 0.05 were considered to indicate statistical significance.

For the analysis of the prediction of outcome after 1 year by the length of stay in ICU, we performed a binary logistic regression analysis and used the baseline data sex, age, and WFNS grade as independent variables. Therefore, we dichotomised the outcome results into poor clinical outcome (mRS ≥ 3) and good clinical outcome (mRS 0-2) (Table 1). Dichotomised outcome was used as the dependent variable (0 = “good outcome”; 1 = “poor outcome”). We analysed the independent effect of the ICU LOS regarding the prediction of a poor clinical outcome (mRS ≥ 3). Moreover, we reported the Nagelkerke R^2^ and Hosmer and Lemeshow test value as quality markers for this analysis.

**Table 1:** Outcome data measured by modified Rankin Scale (mRS).

mRS after 1 year	n	Percentage
0 (no symptoms)	90	54.9%
1 (Minor symptoms)	12	7.3%
2 (Some restriction in lifestyle)	11	6.7%
3 (Significant restriction in lifestyle)	17	10.4%
4 (Partly dependent)	10	6.1%
5 (Fully dependent)	9	5.5%
6 (Dead)	11	6.7%
Good outcome (mRS 0-2)	113	68.9%
Poor outcome (mRS 3-6)	47	28.7%
Total	160	97.6%
Missing	4	2.4%

For binary logistic regression as well as for multiple linear regression we entered all variables en bloc in a single step.

P-values below 0.05 were considered to indicate statistical significance.

## Results

We recorded a total of 250 cases of non-traumatic SAH from 2012 to 2017. We excluded 46 cases of SAH in whom no intracranial aneurysm was detectable. Furthermore, one patient refused consent to participate in the study and therefore was excluded from the database. Of the 203 remaining cases of aneurysmal SAH, we excluded 39 cases of SAH that did not survive the initial time interval until discharge from ICU. Therefore, 164 of 203 patients were available for the analysis regarding prediction of LOS in ICU by complications and interventions. Regarding the analysis of the dependence of ICU-LOS on the outcome after 1 year, 160 of 164 cases were available due to a lack of follow-up data after 1 year in four cases.

Baseline data and outcome data are shown in Tables 1 and 2. Table 3 shows the data regarding complications, interventions, comorbidities, and history of smoking.

**Table 2:** Baseline Characteristics.

Intervention	n	Percentage
Clipping	64	39%
Coiling	100	61%
Sex		
male	63	38.4%
female	101	61.6%
	**Mean**	**Standard deviation**
Age	54.2 y (16-87 y)	13.1
Mean aneurysm size	5.7 mm (2-22 mm)	2.9
**WFNS Grade**	**n**	**Percentage**
Grade I-II	104	63.4%
Grade III	16	9.8%
Grade IV-V	44	26.8%
**Location of aneurysm**	**n**	**Percentage**
ACA/AcoA	63	38.4%
ICA	38	23.2%
MCA	48	29.3%
VA/BA	15	9.1%

**Table 3:** Complications, Interventions, Comorbidities and history of smoking.

Comorbidities	n	%
Pneumonia	66	40.2%
Sepsis	7	4.3%
Hydrocephalus	74	45.1%
Diabetes insipidus	24	14.6%
Decompression	31	18.9%
Delayed Cerebral Ischemia	30	18.3%
Early Cerebral Ischemia	26	15.9%
Hypertension	124	75.6%
Diabetes mellitus	22	13.4%
Hypothyroidism	36	22.0%
History of smoking	67	40.9%
Hypercholesterolemia	44	26.8%
Total	164	100.0%

The mean LOS in ICU was 19 days (median = 17 days; SD = 9.86). The minimum LOS in ICU was 1 day, and the maximum was 76 days.

### Complications and interventions as predictors of length of stay in intensive care units

We performed a multiple linear regression to examine the impact of complications and interventions on the LOS in ICU (see Table 4). Pneumonia, sepsis, hydrocephalus, and delayed cerebral ischemia as well as decompressive craniectomy were statistically significant factors that influenced the LOS in ICU. Sepsis had a very strong impact on LOS (unstandardised β = 9.54), followed by pneumonia (β = 5.11), decompressive craniectomy (β = 5.02), hydrocephalus (β = 4.63), and delayed cerebral ischemia (β = 3.38). Sex, age, and WFNS grade did not reach statistical significance, nor did the complications diabetes insipidus or early cerebral ischemia. The overall regression model (tested by ANOVA/F-test) was significant: F(10, 153) = 12.3, p < 0.0001, R^2^ = 0.45. Adjusted R^2^ was 0.41, and the Durbin-Watson coefficient was 2.23.

**Table 4:** Prediction of Time in intensive care by Complications and Interventions.

Risk factors	β	95% Confidence interval	p
Sex	0.41	−2.042–2.87	0.74
Age	−0.031	−0.13–0.065	0.53
WFNS Grade	0.19	−1.67–2.05	0.84
Pneumonia	5.11	1.75–8.46	0.0031
Sepsis	9.54	3.27–15.82	0.0031
Hydrocephalus	4.63	1.82–7.45	0.0014
Diabetes insipidus	1.04	−2.49–4.58	0.56
Decompression	5.02	1.35–8.70	0.0077
Early Cerebral Ischemia	−1.26	−4.61–2.10	0.46
Delayed cerebral Ischemia	3.38	0.19–6.56	0.038

Linear regression was performed with time in intensive care in days as the dependent variable and sex, age, WFNS grade, pneumonia, sepsis, hydrocephalus, diabetes insipidus, decompression, early cerebral ischemia and delayed cerebral ischemia as independent variables.

### Comorbidities as predictors of length of stay in intensive care unit

In order to predict the LOS in ICU by our recorded comorbidities and history of smoking, we performed a multiple linear regression (see Table 5). None of the comorbidities had a significant impact on the LOS in ICU. Sex and age also did not reach significance. Only the WFNS grade was a significant factor for LOS in ICU with p < 0.0001 and β = 4.66. The overall regression model (tested by ANOVA/F-test) was significant: F(8, 155) = 6.1, p < 0.0001, R^2^ = 0.24. Adjusted R^2^ was 0.20, and the Durbin-Watson coefficient 2.04.

**Table 5:** Prediction of Time in intensive care by Comorbidities.

Risk factors	β	95% Confidence interval	p
Sex	−1.68	−4.613–1.263	0.26
Age	−0.029	−0.146–0.088	0.62
WFNS Grade	4.66	3.021–6.296	<0.0001
Hypertension	−1.07	−4.465–2.336	0.54
Diabetes mellitus	2.59	−1.567–6.745	0.22
Hypothyroidism	3.40	−0.075–6.873	0.055
History of smoking	−2.32	−5.253–0.617	0.12
Hypercholesterolemia	−0.21	−3.542–3.123	0.90

Linear regression was performed with time in intensive care in days as the dependent variable and sex, age, WFNS grade, hypertension, diabetes mellitus, hypothyroidism, history of smoking and hypercholesterolemia as independent variables.

### Length of stay in intensive care unit as a predictor of outcome after 1 year

For this analysis, we used dichotomised outcome values of the mRS scores. Good outcomes were defined as mRS scores of 0‒2. Cases with an mRS Score of 3‒6 were regarded as poor outcomes (see Table 1). For this analysis, 160 of 164 cases of aneurysmal SAH were available. There were four cases of missing outcomes after 1 year. We used a binary logistic regression model to examine the role of LOS in ICU regarding the outcome after 1 year. The Nagelkerke R^2^ value was 0.49, and the Hosmer-Lemeshow test value was 0.41. We adjusted our regression model with the baseline data parameters sex, age, and WFNS grade. All independent parameters (LOS in ICU, age, WFNS grade) had a statistically significant impact on the dependent parameter “outcome after 1 year” except for sex (see Table 6). The LOS in ICU had a significant impact on the outcome after 1 year (OR 1.09). The odds ratio of WFNS grade was 3.72, and for age it was 1.06.

**Table 6:** Time in intensive care and Prediction of Outcome after 1 year.

Risk factors	Odds ratio	95% Confidence interval	p
Time in intensive care	1.09	1.03–1.15	0.0023
Sex	0.75	0.30–1.92	0.55
Age	1.06	1.02–1.10	0.0061
WFNS Grade	3.72	2.23–6.21	<0.0001

Binary logistic regression was performed with dichotomised outcome (0 = “good outcome”; 1 = “poor outcome”) as the dependent variable and time in intensive care, sex, age, and WFNS grade as independent variables.

## Discussion

Aside from the disastrous consequences for the patients and their families, subarachnoid haemorrhage has an important economic impact in terms of cost for the healthcare system. Within the first year, SAH results in far higher costs than reported for ischemic stroke. It is known that initial stay in ICU is one of the main cost-driving factors^5^. Many studies report the impact of different factors on the total hospital LOS, but analyses of the explicit LOS in ICU is rare^3,7,18–20^.

In our analysis, pneumonia (p = 0.0031), sepsis (p = 0.0031), hydrocephalus (p = 0.0014), DCI (p = 0.038), and decompressive craniectomy (p = 0.0077) were the factors that had a significant impact on LOS in ICU. The strongest impact was sepsis (β = 9.54), followed by pneumonia (β = 5.11), decompressive craniectomy (β = 5.02), hydrocephalus (β = 4.63), and DCI (β = 3.38).

The mean LOS in ICU for the significant complications were the following: 36 days (SD = 18.2) with sepsis and 18 days (SD = 8.7) without sepsis, 25 days (SD = 10.6) with pneumonia and 15 days (SD = 6.5) without pneumonia, 24 days (SD = 8.1) with hydrocephalus and 15 days (SD 9.5) without hydrocephalus, 25 days (SD = 12.1) with DCI and 18 days (SD = 8.9) without DCI, and 29 days (SD = 11.2) with decompressive craniectomy and 17 days (SD = 8.1) without decompressive craniectomy. Regarding the WFNS grade, we detected the following mean LOS in ICU: WFNS° I + II 16 days (SD = 7.6), WFNS°III 21 days (SD = 10.2), and WFNS°IV + V 26 days (SD = 11.1).

Frontera *et al*. also detected a significant influence of pneumonia (OR = 2.9; 95% CI = 2.3–3.7; p < 0.001) and bloodstream infection (OR = 1.9; 95% CI = 1.4–2.5; p < 0.001) on the LOS.

The median LOS was 14 days for patients suffering from pneumonia, which was an excess of 7 days (p < 0.001). Patients with bloodstream infections had a median LOS of 12 days (an excess of 4 days) (p < 0.001)^7^. Abulhasan *et al*. reported craniotomy (probability = 1.00; coefficient = 4.54; 95% CI = 2.92-6.16), hospital-acquired infections (including pneumonia and bloodstream infections) (probability = 1.00; coefficient = 9.01; 95% CI = 7.03-10.99), and vasospasm (probability = 1.00; coefficient = 6.23; 95% CI = 4.52-7.94) as significant risk factors for a prolonged LOS in ICU^3^. Dasenbrock *et al*. also confirmed pneumonia (non routine hospital discharge: 86.2%; OR = 3.26; 95% CI = 2.72-3.91; p < 0.001) to be a significant factor regarding the LOS beside urinary tract infections (non routine hospital discharge: 69.8%; OR = 1.51; 95% CI = 1.29-1.77; p < 0.001), CVC-associated infections (non routine hospital discharge: 79.7%; OR = 2.97; 95% CI = 1.13-7.83; p < 0.03), and meningitis/ventriculitis (non routine hospital discharge: 77.7%; OR = 2.37; 95% CI = 1.72-3.26; p < 0.001)^19^. Alaraj *et al*. evaluated 345 SAH patients regarding risk factors of the LOS with 174 patients. In the multivariate analysis, the need for shunt (p < 0.001), clinical vasospasm (p < 0.001), and pneumonia (p = 0.011) were significant factors^18^.

We also analysed the effect of comorbidities and lifestyle on the LOS in ICU. Hypertension, diabetes mellitus, hypothyroidism, hypercholesterolemia, and history of smoking had no significant impact on the LOS in ICU. However, WFNS-grade was a significant predictor of prolonged LOS in ICU in this analysis (see Table 5, β = 4.66). To our knowledge, there is no publication that describes an association of comorbidities and LOS in ICU in detail. Abulhasan *et al*. investigated the effect of respiratory disease on the LOS in ICU and reported a probability of 0.78 (coefficient = 2.43; 95% CI −0.74-5.59). Regarding the total LOS, a probability of 0.99 (coefficient = 13.17; 95% CI = 5.63-20.72) was reported^3^.

In a large national stroke database analysis of all hospital-treated stroke patients in Finland from 1999 to 2007, the LOS increased over time for patients with subarachnoid haemorrhage^21^. The question is whether an increase in the LOS in ICU has a direct effect on the outcome and how strong the impact of LOS on the outcome is. LOS in ICU influences the outcome after 1 year significantly in our binary logistic regression model (p = 0.0023) (Table 6), with an odds ratio of 1.09 per day. When compared directly in our collective, patients with poor outcomes after 1 year had a mean LOS in ICU of 25 days (SD = 11.0), while patients with a good outcome after 1 year had a mean LOS in ICU of 16 days (SD = 7.7). Galea *et al*. compared the median LOS of cases with favourable and unfavourable outcome at discharge and found a significant difference of 14 days in the LOS in the favourable group and 22 days in the unfavourable group (p < 0.001)^8^. Prolonged LOS in ICU comes with a higher risk of complications and interventions, although it is not clear whether the LOS in ICU influences the complications or vice versa.

A different explanation could be that a longer LOS in ICU implies a longer time until patients can undergo full rehabilitation activities. In their study dealing with factors influencing outcomes of cerebrovascular diseases, Ponfick *et al*. showed that every day of in-patient rehabilitation increases the possibility for a favourable outcome across all subgroups (1.9% increase; p < 0.001). Moreover, every day on mechanical ventilation reduces the probability of a beneficial outcome (3.2% reduction; p < 0.05)^22^.

Many other factors have been investigated that determine a prolonged LOS. In a nationwide analysis of 9,635 SAH hospitalizations, Hoh *et al*. showed that clipping compared to coiling was associated with significantly longer LOS (ruptured and unruptured aneurysm: p < 0.0001) and significantly higher total hospital charges (ruptured aneurysm: p 0.0002; unruptured aneurysm: p < 0.0001)^6^. Early worsening after SAH, which occurs in 35% of the patients, was associated with more hospital complications and prolonged LOS and was an independent predictor of death (OR = 12.1, 95% CI = 5.7 to 26.1; p < 0.001) or moderate to severe disability (mRS 4-6, OR = 8.4, 95% CI = 4.9–14.5; p = 0.01) after 1 year in another study^23^. In a systematic review of Mapa *et al*., including 13 studies, hyponatremia was associated with vasospasm and duration of hospitalization, but did not influence mortality^24^. Other studies included more hospital acquired infections and found that urinary tract infections (Frontera *et al*.: OR = 1.7; 95% CI = 1.3–2.1; p < 0.001; Dasenbrock *et al*.: OR = 1.51; 95% CI = 1.29-1.77; p < 0.001) and ventriculitis/meningitis (Frontera *et al*.: OR = 2.1; 95% CI = 1.5–2.9; p < 0.001; Dasenbrock *et al*.: OR = 2.37; 95% CI = 1.72-3.26; p < 0.001) affect the LOS^7,19^. Suarez *et al*. found evidence that the introduction of a neurocritical care team significantly reduced in-hospital mortality (OR = 0.7; 95% CI = 0.5-1.0; p = 0.044) and LOS (neurosciences critical care unit: 4.2 +/− 4.0 days vs. 3.7 +/− 3.4 days; p < 0.001); hospital 9.9 +/− 8.0 days vs. 8.4 +/− 6.9 days; p < 0.0001) without changes in readmission rates or long-term mortality^25^.

The mean time in our ICU was 19 days (4–76 days; SD +/− 9.8) with a median stay of 17 days. This is a relatively high LOS compared to other studies^3,7,8,19,26^. For example, Dasenbrock *et al*. used the data from 7,516 admissions after subarachnoid haemorrhage from the Nationwide Inpatient Sample and reported a median total hospital stay for the population of 16 days (11-23 days)^19^. One reason for the longer LOS in intensive care in our collective might be the high rate of complications with a rate of pneumonia in 40.2% of cases, sepsis in 4.3% of cases, and hydrocephalus in 45.1% of cases, which are critical for the LOS (see Table 3). Dasenbrock *et al*. reported a rate of pneumonia of 23.0% and a rate of sepsis of 5.3%^19^. Galea *et al*. analysed 3,341 patients with SAH and reported a rate of delayed cerebral ischemia of 21.7%, which is comparable to ours (18.3%), a rate of CSF diversion in 34.2%, and CSF sepsis in 5.6%^8^. The cohort also had a large proportion of good grade WFNS patients (WFNS grade I + II 69.5%, III 5.8%, IV + V 24.7%) compared to our collective (see Table 2). In this analysis, the median LOS was 14 and 22 days respectively for patients with favourable and unfavourable outcome^8^.

Another reason for the long LOS in ICU in our study might be the very prudent handling of the transfer from ICU to a ward without surveillance because of the remaining risk of a delayed cerebral ischemia within the first weeks after ictus^27,28^. It is possible that a more rapid transfer from intensive care to other units with adequate surveillance is a factor, which is not fully exhausted in our cohort. Chartrain *et al*. report about a step-down unit transfer protocol for low-risk SAH patients that was safe, feasible, and effective in reducing the LOS in ICU^29^. Moreover logistical limitations (level of support, hospital bed status), may have influenced our results regarding the ICU LOS. With optimised conditions regarding manpower and bed status a further reduction of the ICU LOS might be obtainable. On the other hand, we present the non-optimised conditions, which are reflecting everyday life of the intensive care staff in Central Europe.

Another aspect regarding the total stay of the patients in hospital after SAH are the readmission rates after discharge. Liang *et al*. found readmission rates of 7.8% after 30 days and even 26% after 90 days.

Interestingly up to 14% of the readmissions of the first 30 days were potentially preventable with a large proportion of acute conditions including bacterial pneumonia, and urinary tract infections. Liang *et al*. found an association of a higher readmission rate with diabetes (rate ratio [RR] = 1.09, 95% CI = 1.03-1.15), congestive heart failure (RR = 1.09, 95% CI = 1.00–1.18), and renal impairment (RR = 1.35, 95% CI = 1.13-1.61) whereas discharge home prevented readmission (RR = 0.89, 95% CI = 0.85-0.93)^30^.

### Limitations

Exclusion of patients who did not survive the initial stay in ICU reduced the patient collective from 203 to 164 cases of SAH. This moderate number of cases might be the reason why we could not detect a significant impact of comorbidities on the LOS in ICU. We present data from a single-centre observational study, and our data could only partially be recorded prospectively. Parts of the data regarding complications, interventions, and comorbidities were collected retrospectively.

Timing of a complication or intervention after ictus might be crucial regarding its impact on the length of stay on ICU and outcome after 1 year. In the first days after SAH it is much more difficult for the patient to overcome the negative effects of complications and interventions as it would be after 2 or 3 weeks. The patient has to deal with the direct effects of SAH and with the occurring complication/intervention. This issue might influence the course on ICU significantly regarding the LOS and the outcome after 1 year. Based on a classification of early occurring or late occurring complications/interventions, additional analyses could give a better understanding regarding the impact of distinct interventions/complications in combination with SAH.

Comparability of findings of different studies is crucial in order to bring results in line with the results of existing studies. Hence standardisation is an important aspect. Regarding this issue Stienen *et al*. proposed recommendations dealing with the prioritisation and timing of outcomes and endpoints after aneurysmal SAH^14^. De Oliveira Manoel *et al*. developed common data elements with standardised naming and definitions for hospital course and acute therapies after SAH (including surgical and procedure interventions, rescue therapy for DCI, neurological complications, intensive care unit therapies, medications, and discharge status)^31^.

More powerful prospective randomized trials with high patient numbers and adherence to the recommendations of Liang *et al*. and de Oliveira *et al*. could perhaps detect significant and comparable associations of comorbidities on the LOS in ICU, total LOS, and hospitalisation costs.

Finally, we want to call attention to the fact, that this work is part of a series of investigations which involve comparison of treatment after aneurysmal SAH, outcome and outcome dynamics after SAH and, within this study, LOS in ICU after SAH, where we analysed the same collective in whole or in part^9–11^.

Especially, we want to disclose a related manuscript called “Neurocritical Care Complications and Interventions influence the Outcome in Aneurysmal Subarachnoid Hemorrhage”, which is currently under consideration for publication at a different journal. This work uses the same collective and methods in part, but examines different issues. The related study adds information regarding the impact of complications and interventions during neurocritical care on the outcome after SAH. The main result, conclusion, or implications are not apparent from the other work and vice versa.

## Conclusion

In this observational study, we analysed a collective of patients suffering from subarachnoid haemorrhage who survived until discharge from intensive care. We detected that the complications pneumonia, sepsis, hydrocephalus, DCI, and the intervention decompressive craniectomy were associated with a higher LOS in ICU, which is one of the main driving factors for healthcare costs in SAH patients^5^. However, comorbidities (hypertension, diabetes, hypothyroidism, hypercholesterolemia) and a history of smoking were not significantly associated with a higher LOS in intensive care. The LOS in ICU was significantly although modestly associated with poor outcome after 1 year with an odds ratio of 1.09 for every additional day in ICU. Improvement of strategies regarding prevention and treatment of complications might therefore affect LOS in ICU, hospitalisation costs, and outcome after aneurysmal SAH.

## Data Availability

All data generated or analysed particularly for this study are included in this published article. However, the detailed observational database from 2012 to 2017 pertaining to the 164 cases of aneurysmal SAH is currently not publicly accessible due to further analysis and publication.
